# Analysis of short-term ventilation weaning for patients in spontaneous supratentorial intracranial hemorrhage

**DOI:** 10.1097/MD.0000000000038163

**Published:** 2024-05-17

**Authors:** Yi-Chieh Wu, Hsiang-Chih Liao, Yu-Ching Chou, Peng-Wei Wang, Ming-Hsuan Chung, Wei-Hsiu Liu

**Affiliations:** 1 a Department of Neurological Surgery, Tri-Service General Hospital and National Defense Medical Center, Taipei, Taiwan; 2 b School of Public Health, National Defense Medical Center, Taipei, Taiwan; 3 c Department of Surgery, School of Medicine, National Defense Medical Center, Taipei, Taiwan.

**Keywords:** mechanical ventilation, respiratory weaning, spontaneous supratentorial hemorrhage

## Abstract

Prolonged ventilation is a complication of spontaneous supratentorial hemorrhage patients, but the predictive relationship with successful weaning in this patient cohort is not understood. Here, we evaluate the incidence and factors of ventilation weaning in case of spontaneous supratentorial hemorrhage. We retrospectively studied data from 166 patients in the same hospital from January 2015 to March 2021 and analyzed factors for ventilation weaning. The clinical data recorded included patient age, gender, timing of operation, initial Glasgow Coma Scale (GCS), Intracranial hemorrhage (ICH) score, alcohol drinking, cigarette smoking, medical comorbidity, and the blood data. Predictors of patient outcomes were determined by the Student *t* test, chi-square test, and logistic regression. We recruited and followed 166 patients who received operation for spontaneous supratentorial hemorrhage with cerebral herniation. The group of successful weaning had 84 patients and the group of weaning failed had 82 patients. The patient’s age, type of operation, GCS on admission to the Intensive care unit (ICU), GCS at discharge from the ICU, medical comorbidity was significantly associated with successful weaning, according to Student *t* test and the chi-square test. According to our findings, patients with stereotaxic surgery, less history of cardiovascular or prior cerebral infarction, GCS >8 before admission to the hospital for craniotomy, and a blood albumin value >3.5 g/dL have a higher chance of being successfully weaned off the ventilator within 14 days.

## 1. Introduction

The second most common form of stroke is spontaneous intracerebral hemorrhage (ICH), about 15% to 30% of all strokes. The high morbidity and mortality in ICH patient are also disturbing. The common reasons of nontraumatic ICH include hypertension, vascular anomaly disorders, and coagulopathies like antiplatelet agents. Indeed, the combination of rebleeding, cerebral edema, seizures, increased intracranial pressure (IICP), and hydrocephalus can significantly contribute to neurological deterioration following an initial hemorrhage,^[1,2]^ causing permanent impairment of the patient’s functional abilities in life.

Medical treatment in spontaneous ICH includes blood pressure controlled, anti-epilepsy drugs (AEDs), and hemostasis agent. Management with coagulopathy is necessary in the patient which has coagulopathy or is undergoing anticoagulant medication. Evaluation for operation is important for the patients with evidence of trans-tentorial herniation, intraventricular hemorrhage caused acute hydrocephalus or poor controlled IICP. ICP monitoring insertion or further surgical treatments are considered, for example, the hematoma evacuation revealed some clinical benefits, such as the brain stem prevention, decreased excitotoxicity and neurotoxicity of blood products, cerebral herniation relief and IICP management.^[3]^

For patients exhibiting major midline shift or substantial hematomas on brain computed tomography (CT) scans, or those with unmanageable intracranial hypertension (ICP > 20 mm Hg persisting for longer than 15 min within a 1-h period), surgical intervention involving the removal of intracerebral hematoma may be considered. In cases where first-tier therapies fail to control IICP, a decompressive operation becomes necessary.^[4]^ There are some operative options for decompression. The stereotaxic aspiration of hematoma, craniotomy, and decompressive craniectomy are recommended based on different clinical situations. External ventricular drainage is utilized for acute hydrocephalus, while IICP is addressed in patients with supratentorial hemorrhage and intraventricular hemorrhage.^[5]^ ICP monitoring was inserted for postoperative ICP management. Following the initial surgical intervention, the patient is transferred to an intensive care unit for further monitoring and management. The patient needs to undergo craniotomy due to clinical conditions. Generally, the patient will receive general anesthesia, endotracheal intubation and ventilator assistance.

Patients who have undergone surgical intervention for spontaneous intracerebral hemorrhage (ICH) face a clinical challenge known as prolonged MV (PMV). PMV is characterized by the continued use of mechanical ventilation (MV) for more than 21 days.^[6]^ Long term bed-ridden status may cause more comorbidities. Neurological dysfunction is frequently cited as a primary cause for requiring PMV. This is because brain injury resulting from hemorrhage can lead to reduced levels of consciousness, hypoventilation, and functional disability. PMV has been identified in patients with a high risk for complications during stays in the intensive care unit, longer intensive care unit and hospital stays, high death rates, and higher costs.^[7]^ The risk factors of PMV in patients with spontaneous ICH who have undergone surgical intervention have not been reported. This information could help to resolve the shortage of beds in intensive care units and to use a step-down care procedure for PMV. We retrospectively analyzed data from our patients who received an operation for spontaneous ICH to search for patients who were successfully weaned from MV.

## 2. Materials and methods

**Figure 1.**
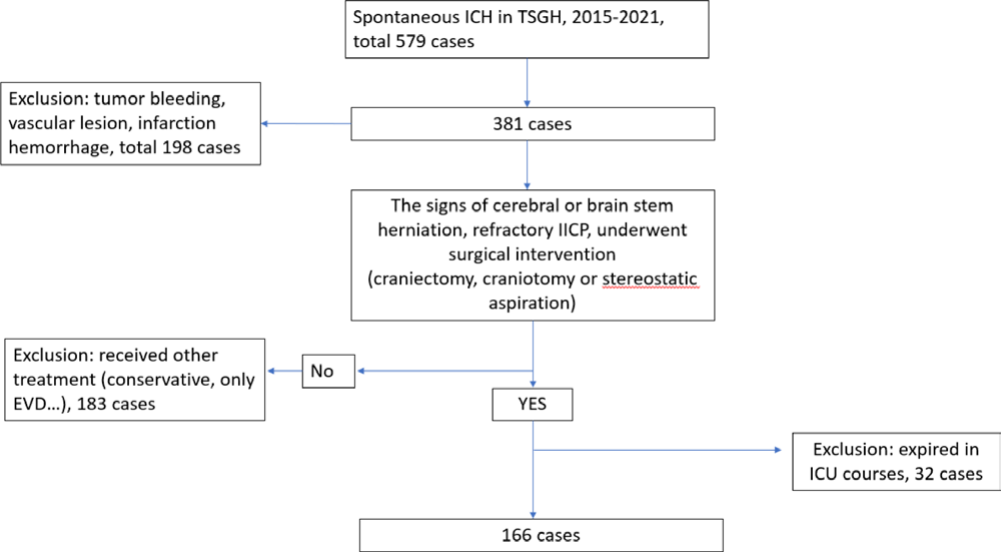
The flow chart of study designs.

Our study focused on patients with spontaneous supratentorial hemorrhage with mass effect who underwent surgical interventions at Tri-Service General Hospital in Taipei, Taiwan, between January 2015 and March 2021. You included 166 patients and excluded those who didn’t initially undergo surgical intervention, had tumor bleeding, or experienced post-infarct hemorrhagic transformation. We exclude the case of infra-tentorial hemorrhage cases due to differences in pathophysiology, pressure dynamics, and neurological symptoms related to hydrocephalus in supra- and infra-tentorial hemorrhage cases. Our study design process included a flow chart (Fig. 1) depicting patient data collection, adhering to the principles outlined in the Declaration of Helsinki. Additionally, the retrospective nature of the study was approved by the Institutional Review Committee at Tri-Service General Hospital. The patients in our study were treated in the intensive care unit and underwent medication for ICP management (head thirty degrees elevation, AEDs, sedative agent, etc). The intracerebral hematoma was nearly total evacuation in the surgery. During the Intensive care unit (ICU) course, the patients were on MV and respiratory training. After cerebral hemorrhage, patients were observed for 1 week, and if there was no sign of neurological deterioration and vital signs were stable, the underwent breathing training to try to wean them off the ventilator. The patient will be excluded from the study if the patient had history of pulmonary diseases, or the patient was suffered from pneumonia during the ICU course.

The patient was sedated for the first 3 days under volume control ventilation. The tidal volume tidal volume and FiO_2_ of ventilation was decided by the ICU doctor and respiratory therapist. The average tidal volume set is 4 to 6 mL/Kg, FiO_2_ is 30% to 35% and the data was adjusted by the patient’s condition. The vital signs and intracranial pressure were closely monitored. If the patient’s vital signs are stable and there is no increase in intracranial pressure, we started to reduce the sedative drugs. If there is no further neurological deficit or respiratory distress, the patient was undergoing breathing training and adjust the pressure control mode to synchronized intermittent mandatory ventilation mode. The spontaneous breathing test (SBT) was started after the clinician assessed the patient’s condition as stable. The ventilator training mode was a SBT or weaning test that comprises a focused assessment of a patient’s capacity to breathe, which is advocated as the best method to ascertain extubating readiness. SBTs are typically designed to assess a patient’s readiness for extubating from MV. This is an important procedure for respiratory training. They usually last between 30 to 60 minutes and involve the patient breathing on their own without full ventilatory support.^[8]^ If the patient’s breathing training is not satisfactory and cannot be successfully released from the respirator after 1 week of training (after 14 d of hospitalization in the intensive care unit), the patient is transferred to the respiratory care center for continuous breathing training and may receive tracheostomy in the respiratory care center in a future surgery. Therefore, we target a short-term ventilator weaning period of 14 days.^[9]^ No further connection to ventilator more than 120 hours is the definition of weaning in tri-service general hospital.

The clinical data included patient age, gender, initial Glasgow Coma Scale (GCS), intraventricular hemorrhage, alcohol drinking, cigarette smoking, medical comorbidities (history of cancer, old stroke, hemodialysis, heart valve diseases, anticoagulant agent usage, type 2 diabetes mellitus), and albumin levels (which we check at admission to the ICU). Univariate analysis was utilized to explore the correlation between each explanatory factor and the outcome of ventilation weaning post-ICH. Student *t* test and either the chi-squared test or Fisher’s exact test were employed for continuous and categorical data, respectively. Multivariate analysis was conducted using logistic regression as previously described to identify predictors. A significance level of *P* ≤ .05 denoted a statistically significant difference. This study and experimental design comply with the regulations of the Human Research Ethics Committee, ensuring the respect of patients’ rights and safety throughout the research process.

## 3. Results

A total of 166 patients who received surgical intervention for spontaneous ICH were included in our study, including 58 women and 108 men. Patient age ranged from 24 to 89 years (mean: 57.8 yrs). The spontaneous ICH volume had a mean value of 59.2 mL. The patient’s GCS on admission to the ER ranged from 3 to 14 (mean: 7.8), and the GCS on discharge from the ICU ranged from 3 to 15 (mean: 10.7). The hospitalization time in the ICU ranged from 4 to 79 days (mean: 17.0 d). The duration of removal of the endotracheal tube ranged from 4 to 46 days (mean: 10.3 d). The group of successful weaning had 84 patients and failed had 82 patients.

The weaning from MV was not significantly associated with patient gender, smoking habit, drinking alcohol, intraventricular hemorrhage, and volume of hematoma. The patient’s age, type of operation, GCS on admission to the ICU, GCS at discharge from the ICU, Albumin level in the first 3 days after admission, medical comorbidity was significantly associated with successful weaning. The distribution of demographic and clinical characteristics is presented in Table 1.

**Table 1:** The distribution of demography and clinical characteristic.

Variables	Mean	Minimal	Maximal	
Age (yrs)	57.8	24.0	89.0	
Hematoma volume (mL)	59.2	19.6	158.5	
GCS on admission	7.8	3	14	
GCS on discharge From ICU	10.7	3	15	
ICU days	17.0	4.0	79.0	
Time to remove ETT	10.3	2.0	46.0	
Albumin	3.4	2.2	4.3	
CRP	3.5	0.1	9.4	
	Failed weaning (n = 82)	Success weaning (n = 84)	χ^2^ or Z	*P* value
Sexual, n (%)			0.861	.354
Female	32 (39.0)	26 (31.0)		
Male	50 (61.0)	58 (69.0)		
Age, n (%)				.014
<65	49 (59.7)	67 (79.7)		
≥65	33 (40.3)	17 (20.3)		
Operation, n (%)			6.503	.038
Craniectomy	48 (58.6)	36 (42.9)		
Craniotomy	28 (34.1)	32 (38.1)		
Stereotactic	6 (7.3)	16 (19.0)		
Blood volume, n (%)			2.442	.295
<30 mL	14 (17.1)	13 (15.5)		
30–80 mL	48 (58.5)	58 (69.0)		
>80 mL	20 (24.4)	13 (15.5)		
Intraventricular hemorrhage, n (%)			5.408	.020
Yes	48 (58.5)	33 (39.3)		
No	34 (41.5)	51 (60.7)		
GCS in admission, n (%)			26.202	<.001
≤8	70 (85.4)	39 (46.4)		
>8	12 (14.6)	45 (53.6)		
GCS at discharge from ICU, n (%)			29.226	<.001
≤8	26 (31.7)	0 (0)		
>8	56 (68.3)	84 (100)		
Alcohol, n (%)			0.002	.968
Yes	30 (36.6)	32 (38.1)		
No	52 (63.4)	52 (61.9)		
Smoking, n (%)			1.640	.200
Yes	19 (23.2)	28 (33.3)		
No	63 (76.8)	56 (66.7)		
Medical comorbidity, n (%)			13.923	.001
≤1	21 (26.9)	39 (47.0)		
2	25 (32.1)	31 (37.3)		
≥3	32 (41.0)	13 (15.7)		
Hemodialysis, n (%)				.115
Yes	5 (6.1)	1 (1.2)		
No	77 (93.9)	83 (98.8)		
Hypertension, n (%)			0.015	.904
Yes	67 (81.7)	67 (79.8)		
No	15 (18.3)	17 (20.2)		
Cardiovascular diseases, n (%)			11.797	.001
Yes	32 (39.0)	12 (14.3)		
No	50 (61.0)	72 (85.7)		
Old cerebral infarction, n (%)			7.531	.006
Yes	25 (30.5)	10 (11.9)		
No	57 (69.5)	74 (88.1)		
Type 2 diabetes mellitus, n (%)			1.548	.213
Yes	20 (24.4)	13 (15.5)		
No	62 (85.6)	71 (84.5)		
Autoimmune diseases, n (%)			0.002	.967
Yes	6 (7.3)	5 (6.0)		
No	76 (92.7)	79 (94.0)		
Hyperlipidemia, n (%)			0.009	.925
Yes	23 (28.0)	22 (26.2)		
No	59 (72.0)	62 (73.8)		
Chronic lung diseases, n (%)			0.750	.387
Yes	10 (12.3)	6 (7.1)		
No	72 (87.7)	78 (92.9)		
Cancer, n (%)			0.002	.962
Yes	7 (8.5)	7 (8.3)		
No	75 (91.5)	77 (91.7)		
Albumin, n (%)			139.516	<.001
<3.5 g/dL	81 (98.8)	5 (6.0)		
≥3.5 g/dL	1 (1.2)	79 (94.0)		

CRP = C-reactive protein, ETT = endotracheal tube.

We used univariate and multivariate logistic regression analysis to evaluate the data presented in Table 2. Patients who received stereotactic aspiration were likely to have a higher incidence of successful weaning compared to those who received craniectomy or craniotomy (odds ratio [OR] 3.96, 95% confidence ratio [CI] 1.03–15.26). Patients who had more medical comorbidities were associated with PMV, especially those with involvement of more than 3 systems (OR: 0.16; 95% CI: 0.06–0.48). GCS >8 on admission to the ICU was associated with successful weaning (OR: 19.32; 95% CI: 6.42–15.26). More medical comorbidity (≥3, especially cardiovascular diseases and history of stroke) was significant difficulty associated with ventilation training.

**Table 2:** Multivariate logistic regression analysis.

Variables	OR (95% CI)	*P* value
Age (≥65 vs <65)	0.15 (0.05–0.47)	.001
Sex (Male vs Female)	1.38 (0.55–3.44)	.494
Operation (Craniectomy)	1.00	(ref)
Operation (Craniotomy)	1.05 (0.45–2.45)	.918
Operation (Stereotaxic)	3.96 (1.03–15.26)	.046
Initial GCS (>8 vs ≤8)	19.32 (6.42–58.17)	<.001
Total comorbidity≤1	1.00	(ref)
Total comorbidity = 2	0.64 (0.25–1.64)	.352
Total comorbidity≥3	0.16 (0.06–0.48)	.001

CI = confidence interval, OR = odds ratio, ref = reference group.

## 4. Discussion

Our study was conducted to determine the predictors for ventilator weaning in patients who suffered from spontaneous ICH. We found at least 5 predictors for highly successful weaning in these cases: age <65 years, receiving stereotactic aspiration, less medical comorbidity, GCS >8 on admission to the ICU, and albumin level. These had statistical significance in predicting the incidence of weaning from ventilation.

### 4.1. Age of successfully weaned patients

In our study, 116 patients (69.9%) were younger than 65 years, and 50 patients (30.1%) were older. This result demonstrates that the percentages of successful attempts decrease with increasing age. One of the reasons for the poor performance of ventilator training in the elderly could be that older people have more medical problems (average number comorbidities: 2.3). According to our study statistics, those with a history of cardiovascular disease who received anticoagulants or had a previous cerebral infarction performed worse on ventilator training disengagement, with 24 (48%) of 50 patients over 65 years old having a heart attack. Of those with a history of vascular disease who received anticoagulant treatment, 13 (26%) had a previous cerebral infarction, and the proportion of patients with a history of cardiovascular disease and previous cerebral infarction was lower in the group of patients younger than 65 years old (17.2% had a history of cardiovascular disease, and 18.1% had a previous cerebral infarction).

Patients with a cardiovascular disease background tend to have limited success during breathing training. Adverse cardiovascular reactions during MV and the process of weaning off it encompass changes in hemodynamics and stability, myocardial ischemia, dysfunction in autonomic regulation, and irregularities in cardiac rhythm.^[10]^ Patients with a prior cerebral infarction combined with neurological impairment are more serious. Respiratory system function disturbances and complications impacting the respiratory system frequently occur poststroke. The characteristics of these disorders vary based on the extent and location of neurological damage. Changes in breathing regulation, respiratory mechanics, and breathing rhythm are prevalent and can result in gas-exchange irregularities or necessitate MV.^[11]^ These reasons and others may cause patients with acute ICH to have no way to be weaned off the ventilator for 2 weeks after surgery.

### 4.2. Glasgow coma scale score on admission to the intensive care unit and Glasgow coma scale score at discharge from the intensive care unit

On admission to the ICU, 57 patients (34.3%) with GCS more than 8 and 109 patients (65.7%) with GCS less than 8 were noted. At discharge from the ICU, 140 patients (84.3%) with GCS more than 8 and 26 patients (15.7%) with GCS less than 8 were noted. Difficulty of follow-up ventilator training arises from unfavorable factors in patients with ICH such as admission to the hospital with a GCS score of 8 or less before craniotomy combined with severe neurological impairment, including cranial nerve damage, limb weakness, labored breathing, etc. A simplified clinical score was created to evaluate cough, swallowing, gag reflex, and neurological status in a preliminary group of brain injury patients. This score was then tested internally for validation.^[12]^ Compared with the group with GCS >8 points, there are fewer symptoms of nerve damage, the difficulty of follow-up breathing training is lower, and there is a higher chance being cured after craniotomy. They are also weaned off respirator within 2 weeks. Based on research on intubation with GCS < 8, we arrange admission to a respiratory care ward or a tracheostomy to facilitate follow-up care for patients whose GCS is still less than or equal to 8 after 2 weeks of treatment in the intensive care unit.

### 4.3. The surgical intervention and clinical management of successfully weaned intracranial hemorrhage prolonged mechanical ventilation patients

In our study, 84 patients (50.6%) received craniotomy with removal of hematoma, 60 patients (36.1%) received decompressive craniectomy, and 22 patients (13.3%) received stereotactic aspiration of the hematoma. Compared with patients who underwent decompressive craniectomy, patients who underwent craniotomy had a higher proportion of short-term weaning (53.3%), and those who underwent stereotactic aspiration had an even higher proportion (72.7%). Decompressive craniectomy with hematoma evacuation can be beneficial for comatose patients with substantial midline shift and large hematomas seen on brain CT scans, severe neurological deficit or refractory intracranial hypertension (IICP) who do not improve with initial therapies (defined as intracranial pressure [ICP] > 20 mm Hg for > 15 min within an hour). This group has severe initial neurological impairment, resulting in poor postoperative complications and patient prognosis. Therefore, the performance of breathing training is relatively poor. It is not ideal for the other 2 groups, and skull defects may be combined with sink flap syndrome.^[13]^

In sink flap syndrome, motor symptoms like hemiparesis are commonly observed, while a range of cognitive symptoms such as bradypsychia, speech fluency issues, naming difficulties, aboulia, dysphasia, dyspraxia, and astereognosis have also been reported. Changes in blood flow regulation and cerebral glucose metabolism due to craniectomy may play a role in these symptoms, supported by studies using CT scans, CT perfusion imaging, and Xenon CT, suggesting a vascular mechanism.^[14]^ These symptoms may increase the difficulty of clinical breathing training.

### 4.4. Nutrition condition of successfully weaned patients

In medical patients undergoing weaning attempts, both cough strength and albumin levels are identified as independent risk factors for reintubation. The likelihood of reintubation increases with multiple SBT attempts, weak cough, and low albumin levels in these patients.^[15]^ This study provides information for clinical practitioners for the consideration of patient extubation. In our study, 80 patients had albumin levels above 3.5 g/dL, and 86 patients had albumin levels below 3.5 g/dL. Those with albumin levels greater than or equal to 3.5 g/dL had a significantly higher proportion of short-term ventilator release. Therefore, if a patient’s initial albumin level is <3.5 g/dL, perhaps the patient should be cared for in an intensive care unit. Additional supplementation of albumin during the period could increase the chance of the patient being released from the respirator, which is also a direction that we could continue to study in the future.

### 4.5. Limitations of our study

Acknowledging the limitations of your study as retrospective, nonrandomized, and conducted in a single center is important for providing context to your research findings. These limitations can influence the generalizability and robustness of the study’s conclusions. Not collecting essential variables like laboratory data, respiratory parameters, and scores like Acute Physiology and Chronic Health Evaluation II can indeed lead to information bias and limit the depth of analysis in your study. It’s crucial to acknowledge these limitations when interpreting and discussing your research findings. Whether respiration-related indexes cause respiratory muscle training and detachment bias is also a topic that we need to study in depth in the future. Furthermore, the number of cases of cerebral hemorrhage combined with chronic lung disease, autoimmune disorder, cancer, and hemodialysis was relatively small, which may have caused bias in the clinical analysis.

## 5. Conclusion

Breathing training and ventilator detachment among patients after spontaneous ICH are closely related to the prognosis of neurological function. Patients who can be released from the ventilator as soon as possible can receive rehabilitation therapy and other adjuvant therapy earlier to achieve better daily performance.^[16]^ According to our findings, patients with stereotaxic surgery, less history of cardiovascular or prior cerebral infarction, GCS >8 before admission to the hospital for craniotomy, and a blood albumin value >3.5 g/dL have a higher chance of being successfully weaned off the ventilator within 14 days.

## Author contributions

**Conceptualization:** Yi Chieh Wu, Wei-Hsiu Liu.

**Data curation:** Yi Chieh Wu, Wei-Hsiu Liu, Hsiang Chih Liao, Yu-Ching Chou, Wang Peng Wei, Chung ming hsuan.

**Formal analysis:** Yi Chieh Wu, Wei-Hsiu Liu, Hsiang Chih Liao, Yu-Ching Chou, Wang Peng Wei, Chung ming hsuan.

**Investigation:** Yi Chieh Wu, Hsiang Chih Liao, Yu-Ching Chou.

**Project administration:** Yi Chieh Wu, Yu-Ching Chou.

**Resources:** Yi Chieh Wu, Yu-Ching Chou.

**Software:** Yi Chieh Wu.

**Writing – original draft:** Yi Chieh Wu.

**Writing – review & editing:** Wei-Hsiu Liu.
